# Interpreting the Possible Ecological Role(s) of Cyanotoxins: Compounds for Competitive Advantage and/or Physiological Aide? 

**DOI:** 10.3390/md11072239

**Published:** 2013-06-27

**Authors:** Aleicia Holland, Susan Kinnear

**Affiliations:** Centre for Environmental Management, Central Queensland University, Bruce Highway, Rockhampton, QLD 4700, Australia; E-Mail: a.holland@cqu.edu.au

**Keywords:** anatoxin-a, allelopathy, cyanobacteria, cyanoprokaryotes, cylindrospermopsin, microcystin, saxitoxin

## Abstract

To date, most research on freshwater cyanotoxin(s) has focused on understanding the dynamics of toxin production and decomposition, as well as evaluating the environmental conditions that trigger toxin production, all with the objective of informing management strategies and options for risk reduction. Comparatively few research studies have considered how this information can be used to understand the broader ecological role of cyanotoxin(s), and the possible applications of this knowledge to the management of toxic blooms. This paper explores the ecological, toxicological, and genetic evidence for cyanotoxin production in natural environments. The possible evolutionary advantages of toxin production are grouped into two main themes: That of “competitive advantage” or “physiological aide”. The first grouping illustrates how compounds produced by cyanobacteria may have originated from the need for a cellular defence mechanism, in response to grazing pressure and/or resource competition. The second grouping considers the contribution that secondary metabolites make to improved cellular physiology, through benefits to homeostasis, photosynthetic efficiencies, and accelerated growth rates. The discussion also includes other factors in the debate about possible evolutionary roles for toxins, such as different modes of exposures and effects on non-target (*i.e.*, non-competitive) species. The paper demonstrates that complex and multiple factors are at play in driving evolutionary processes in aquatic environments. This information may provide a fresh perspective on managing toxic blooms, including the need to use a “systems approach” to understand how physico-chemical conditions, as well biological stressors, interact to trigger toxin production.

## 1. Introduction

### Cyanobacteria and Their Toxins

**Figure 1:**
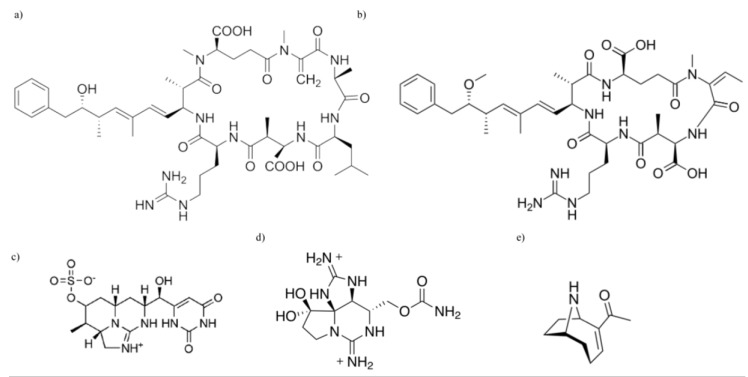
Chemical structures of cyanotoxins: (**a**) microcystin (MC); (**b**) nodularin (NOD); (**c**) cylindrospermopsin (CYN); (**d**) saxitoxin (STX); and (**e**) anatoxin-a.

The cyanobacteria, or blue-green algae, are photosynthetic microorganisms commonly found in a diverse range of aquatic and terrestrial environments including Antarctic lakes, thermal springs, arid deserts, and tropical acidic soils [1,2]. Typically, however, it is the toxin-producing blooms of these species that are of greatest interest and concern to humans, especially where these appear in tropical, subtropical, and temperate fresh and marine waters used for recreational or drinking water purposes. Cyanotoxins are recognized as toxic to a wide variety of trophic levels, including organisms such as bacteria, protozoans, zooplankton, fish, birds, and mammals [3,4,5]. As a group, the cyanotoxins have a wide array of chemical structures, triggers for production and modes of toxicity; and the toxicity of these compounds varies amongst different producer species (Figure 1). 

The literature on toxin production by the cyanobacteria is already large, and continues to grow as new toxins are identified, and new tools and techniques are developed for their study. The latter have been largely focused on the rapid detection through the use of molecular and genetic methods, particularly in field applications, with the objective of improving risk management in drinking water supplies. Despite these advances, several authors have acknowledged that the ecophysiology and broader role of toxin production by cyanobacteria remains very unclear [6,7,8]. This information is useful not only in the sense of understanding the ecology and biology of cyanobacterial species, but also through the potential to provide insights into how they may be best managed in terms of drinking water, recreational water, and environmental health risks. 

## 2. Determining Possible Ecological Roles

### 2.1. Toxins as Secondary Metabolites for Competitive Advantage

Since cyanotoxins began appearing in scientific literature, they have generally been accepted as “secondary metabolites” [9]. By definition, secondary metabolites are those compounds not used by an organism for primary metabolism [10]. Hormones, antibiotics, and allelochemicals are each examples of secondary metabolites. The formation of secondary metabolites is subject to general physiological control that responds to environmental factors. In plants, secondary metabolites, such as alkaloids, were once regarded as waste material discarded from metabolism, which accumulated in tissues simply due to the absence of an appropriate excretory system. However, this view is no longer generally accepted as such substances were later assigned a beneficial role such as controlling predation (e.g., through poor palatability) [11]. 

The presence of a regulatory mechanism associated with the production of secondary metabolites suggests that the evolution of these compounds is based in their ability to confer benefit on the cell [11,12], as opposed to being truly “accidental” and/or “leftover” compounds. Some authors have suggested that microbial secondary metabolites evolved during periods of ecological disequilibrium, where they were able to provide a distinct competitive advantage [13], but which may or may not be relevant in modern aquatic environments. For example, compounds formed in response to rapid change environments may still confer benefit in the form of competitive advantage. It is also possible that cyanobacterial secondary metabolites can in fact provide multiple functions, as this is known from other microbial species (e.g., in antibiotic production). Thus, they could simultaneously offer functions in both primary and secondary metabolism. In this light, the labelling of cyanotoxins as “secondary metabolites” has become blurred, because there appears to be evidence for of the potential for their role in primary metabolism—either currently, or in the past. 

### 2.2. Toxins for Core Physiological Functions

Since cyanotoxins affect numerous aquatic and terrestrial organisms, it is tempting to conclude that toxin production predominantly occurs to offer a defence against grazing and/or to reduce resource competition. However, it is also possible that the primary function(s) of cyanotoxins are unrelated to their toxic properties [14]. Given that toxin production by cyanobacteria has been retained over long evolutionary periods, and despite the metabolic costs of production, it seems highly likely that these compounds have important biological function(s) [15]. For example, recent studies examining cyanotoxin production have found close links between these compounds and certain physiological functions that may be considered part of the primary metabolism of the cell [7]. A number of other physiological roles have been speculated for cyanotoxins, such as cell signalling, nutrient uptake, iron scavenging, maintenance of homeostasis, and protection against oxidative stress [7,16,17,18,19].

## 3. Exploring the Evidence

### 3.1. Environmental Triggers for Toxin Production

#### 3.1.1. Abiotic Factors

Cyanotoxin production is dependent on a number of environmental conditions: Predominantly, these include nutrient concentration, light intensity, and temperature. Nutrients have been shown to influence the production of various toxins, across several cyanobacterial genera. For example, nitrogen-limited environments are generally associated with elevated levels of cylindrospermopsin (CYN), microcystin (MC), anatoxin-a and nodularin, with each of these being produced by nitrogen-fixing cyanobacteria [8,20]. Conversely, toxin production in non-nitrogen fixing cyanobacteria, such as *Microcystis* and *Planktothrix*, has been shown to peak with high concentrations of nitrogen [8,21]. This presents a scenario whereby the presence of N can contribute to both increased and decreased MC production, depending on the genera. The findings related to phosphorous also demonstrate the importance of this nutrient to toxin production: Basic *et al.* [22] reported a decrease in CYN production under phosphorous limitation, and CYN production was shown to increase with phosphorus concentrations in other studies [23,24]. 

Light intensity is also a critical factor influencing the production of cyanotoxins. For example, the highest CYN concentrations are not found at the light intensities that are optimal for growth (50–100 μmol·m^−2^·s^−1^), but instead, from intensities outside this range [20]. Dyble *et al.* [25] also reported that maximum CYN production occurs at supra-optimal light intensities, with the highest CYN produced at the highest intensity. For microcystin, the transcription of two genes responsible for toxin production was shown to be influenced by light quality: Here, maximum transcription rates were recorded at high light intensities and under red light, whereas blue light caused decreased transcription [26].

Like light, temperature has a fundamental relationship with cyanotoxin production, yet here, the physiology seems more closely linked with the regulation of growth rates. The highest CYN concentrations, for example, are often found at temperatures that would be considered sub-optimum for cell growth, with maximum CYN reported at 20 °C and production ceasing at temperatures exceeding 35 °C. There is a negative correlation between growth rate and the rate of CYN produced [20], presumably resulting from the metabolic trade-offs involved with reproduction *vs.* toxin production. Anatoxin-a production has also been shown to be highest at 20 °C [27], whereas maximum production of MC and nodularin has been reported to occur between 18 and 25 °C [8]. 

The pH of water may also influence toxin production. Van der Westhuizen *et al.* [28] in Jaiswal *et al.* [29] reported that higher MC production occurred at pH values above and below the optimum growth brackets for *M. aeruginosa*, however, other studies have found no direct effect of pH on MC production [29].

#### 3.1.2. Biotic Factors

The presence of a competitor or predator clearly influences the level of production of many cyanotoxins, but the extent to which this occurs is not consistent amongst toxins, even when the same producer species is involved [30]. For example, production of anatoxin-a by *Anabaena flos-aquae* was shown to increase in the presence of the green alga *Chlamydomonas reinhardtii*, whereas the production of MC was totally inhibited [31]. Differences in MC production by *Microcystis* were also shown to increase upon direct or indirect (chemical cues from feeding) exposure to grazers such as microcrustaceans and phytoplanktivorous fish [32,33,34].

### 3.2. Toxicity for Competitive Advantage

#### 3.2.1. Grazing Defence

Cyanobacterial toxins are widely believed to have evolved in response to grazing pressure: Here, the toxin production provides producer species with a competitive edge over their non-toxic counterparts. Numerous studies have shown that cyanotoxins are toxic to zooplankton; indeed, some zooplankters actively avoid them [35,36,37]. For example, Demott *et al.* [37] observed that low concentrations of MC inhibited feeding rate of *Daphnia pulicaria*; however, when this microcrustacean was placed in toxin-free water, the feeding rate quickly recovered. The authors thus suggested that the toxin had evolved as a chemical defence against grazers [37]. The toxin-producing *Anabaena flos-aquae* has been shown to decrease lifespan, fecundity, and population growth rate of the rotifers *Brachionus calycijlorus* and *Synchaeta pectinata* [36]. The production of MC by *Microcystis* was also shown to increase in response to direct cues from exposure to grazers such as microcrustaceans and phytoplanktivorous fish or indirect chemical cues from feeding [32,33,34]. CYN has been shown to be toxic to a number of aquatic organisms including brine shrimp *Artemia salina* [38], *Daphnia magna*, and *Daphnia galeata* [39]. Each of these examples supports the proposition that toxin production evolved as a defence mechanism against grazing. However, a study by Van Gremberghe *et al.* [40] found that this phenomenon was strain specific; and in general, exposure to infochemicals from *Daphnia* sp. have a weak influence on toxin production. Wilken *et al.* [41] also concluded that direct and indirect exposure to a protozoan grazer did not cause an increase in MC. This presents some difficulty in concluding that grazing inhibition is the sole—or even predominant—reason for the evolution of cyanotoxin.

To further add to the complexity of this issue, recent advances in the molecular field have shown that the genes involved in the production of some toxins may predate the metazoan lineage [42,43,44], hence confounding any notion that toxin production was originally triggered by grazing pressure. For example, molecular analysis on the MC synthetase genes have shown that these were present in the ancient ancestor of cyanobacteria, some 1600–2000 million years ago [43]. The *nda* gene, which codes for the nodularin was also shown to originate from an ancient ancestor [44]. Recent determination of the genetic basis for saxitoxin (STX) has revealed that the STX gene clusters involved in the production of this toxin appear to have been present early in the divergence of the Nostocales, at least 2100 million years ago [42]. The oldest fossils of filamentous akinete-forming cyanobacteria have been dated to, between, 1600–2000 million years of age [45]. Given that most of the cyanobacteria, which produce CYN form akinetes, like MC and STX, it is likely that the genes responsible for CYN production were present in their ancient ancestor, which predates the divergence of metazoans which occurred approximately 1576 million years ago [43].

Whilst the molecular evidence strongly suggests that the evolution of genes for toxin production pre-dated the existence of metazoans, this does not eliminate all possibility for evolution of cyanotoxins as a grazing defence mechanism: Protozoans may have been able to provide sufficient pressure [41]. In many systems, the biomass of protozoans often exceeds that of metazoans, in fact, protozoans are often the most important grazers on phytoplankton [46]. Unsurprisingly, a number of studies have demonstrated adverse effects of cyanotoxins on protozoans: For example, CYN is toxic to the amoeba *Naegleria lovaniensis* [47], and the protozoans *Spirostomum ambiguum* and *Tetrahymena termophyla* are both sensitive to MC [48]. Conversely, a number of protozoan grazers are known to actively feed and grow on toxic cyanobacteria [46,49]. The study by Fabbro *et al.* [49] found that the ciliate *Paramecium caudatum* was able to successfully graze on the CYN producer *Cylindrospermopsis raciborskii*; hence the probable reason that this ciliate regularly co-occurs with environmental blooms of that alga. However, in the same study, variation in toxin production was observed between the straight and circular forms of *C. raciborskii* when these were challenged by feeding of *P. caudatum*, with no effect recorded from the strain having circular morphology. A more recent study also demonstrated that *Microcystis aeruginosa* was unable produce MC in response to direct and indirect exposure to a protozoan grazer [41]. This suggests that whilst toxin production is likely to be linked with defence against protozoan grazing in some species and/or morphological strains blue-greens, this is unlikely to be the case for all species, which means alternative explanations are required.

Furthermore, the argument against “toxins as a grazing defence” is made stronger when the overall cost of toxin production is considered: When judged entirely by the metabolic balance sheet, cyanotoxins may be a poor choice for establishing a defence mechanism. For example, many algae—including those that produce toxins feature potentially more cost-effective methods of reducing grazing, such as the production of mucous, the formation of large colonies as observed in species of *Microcystis* sp., or differentiation into cell morphologies that are less amenable to grazing (such as coiled *Cylindrospermopsis*, as mentioned above). 

A further complication against the “grazing defence” argument is the multiple examples of species that produce and retain toxin largely in the intracellular form: This is particularly true of most MC producers, although not the case for CYN (where large proportions of the toxin occur in the extracellular environment). Pragmatically, the “grazing defence” argument is much weaker for species that rely on intracellular toxin, as the beneficial effect of the toxin (to deter grazing) is only imparted once the cell has already been consumed. A possible counterargument to this would be that intracellular toxin acts to reduce palatability, thus increasing the fitness of a clonal colony, leading to genetic advantage for the strain and/or population. Questions about the role of toxin in reducing grazing pressure are also raised when the variety of target animals for cyanotoxins is considered. For example, invertebrates (e.g., aquatic snails) appear much less sensitive to the toxicity of CYN compared with vertebrates (e.g., tadpoles, fish), despite the snails representing a similar (or greater) threat to that producing species in terms of grazing pressure [50]. 

#### 3.2.2. Allelopathy

Allelopathy can be defined as the inhibition of growth of one organism by the release of chemicals by another, and which typically confers benefit in terms of reduced resource competition. Toxin-producing cyanobacteria constantly compete against other cyanobacteria, algae and macrophytes for light, nutrients and space. Thus, an ecological role for cyanotoxins as an allelopathic chemical against other algae and plants has been proposed, and several studies have provided evidence for this hypothesis. Singh *et al.* [51] showed that purified Microcystin-LR (MC-LR) extracted from *Microcystis aeruginosa* had a negative effect on the growth of several green algae and cyanobacteria. Kearns and Hunter [52] also showed that MC and anatoxin-a both inhibit the motility of the green alga *Chlamydomonas reinhardtii*. The cyanobacterium *Synechococcus elongatus* was also shown to be inhibited by the presence of the Microcystin-RR (MC-RR), possibly due to inhibition of photosynthesis by the toxin [53]. MC-LR has also been shown to exert inhibitory effects on aquatic plants, such as *Ceratophyllum demersum*, with the toxin inhibiting growth, morphology, and photosynthesis at environmentally relevant concentrations (5 μg·L^−1^) [54,55]. Inhibitory effects of MC-LR were also reported for the growth and morphology of the aquatic plant *Spirodela oligorrhiza*, however, the test concentration used were far higher than those reported from the field [56]. Similary, anatoxins have also been shown to have negative effects on aquatic plants, but only at concentrations exceeding environmental relevance [57]. Cell and cell-free extracts of *C. raciborskii* were linked with allelopathic effects on two green algae, *Coelastrum sphaericum* and *Monoraphidium contortum*, the cyanobacterium *Microcystis wesenbergii*, and the diatom *Navicula* sp. [58]. Growth of *Lactuca sativa*, *Phaseolus vulgaris*, *Pisum sativum*, and *Solanum lycopersicum* was also shown to be affected by extracts of two strains of *C. raciborskii*, although germination was not significantly inhibited [59]. The influence of cyanotoxins on nutrient dynamics on other organisms can also be considered a form of indirect allelopathy [60].

In contrast to the above, MC-LR and CYN at ecologically relevant concentrations were recently shown to lack any allelopathic effects on some species of phytoplankton [61]. This study showed that cyanobacterial crude extracts induced more pronounced effects on growth rates compared with pure toxins, with stimulatory effects noted for extracts containing MC-LR and CYN at 0.025–2.5 mg·L^−1^. *Hydrilla verticillata* was also shown to display significant growth stimulation and redistribution of plant resources in conjunction with exposure to the whole-cell extracts of *C. raciborksii*, containing up to 400 μg·L^−1^ CYN [62].

Considering the two sets of evidence above, the notion that allelopathy is dominant in the ecological role of cyanotoxins remains under debate. A number of authors have suggested that toxins themselves may not have allelopathic effects, but instead work synergistically with other cellular compounds: This line of thought has been prompted by the observations that very high toxin concentrations are generally required to exert inhibitory effects, and because cellular extracts containing toxins are often more active than purified toxin [30,61,63]. For example, Babica *et al.* [63] noted that only a limited number of studies illustrated an allelopathic effect on phototrophic organisms at environmentally relevant concentrations of MC, and concluded that the ability of MC to work as an allelopathic chemical seemed unlikely. Berry *et al.* [64] also noted that typical environmental concentrations of MC are below 10 μg/L, whereas the studies demonstrating inhibitory effects on photoautotrophs were conducted using concentrations greater than 100 μg·L^−1^ MC. There is also a lack of experimental results on allelopathy at pre-bloom cell densities [65], with the few existing studies of low cell concentrations failing to demonstrate any effects; and a lack of studies to integrate laboratory and field observations with respect to establishing allelopathic effects [66]. Allelopathic tendencies are also considered much reduced in those toxins, which present intracellularly (such as MC) [66]. 

### 3.3. Toxic Compounds as Physiological Aides

#### 3.3.1. Assistance in Nutrient Uptake

The availability of nutrients is recognized as a major limiting factor for the proliferation of phytoplankton in freshwater. Despite this importance, there seems to be a shortage of studies about the effect nutrient concentrations have on toxin production, and whether toxins may play an ecological role in improving access to nutrients [22]. One exception to this is the information about the role of toxins in mediating alkaline phosphatases (APase) secretion by other organisms, which could potentially offer ecological advantage to the toxin-producer. For example, it has been demonstrated that when phosphate is limited, inorganic phosphate is taken up from the environment by autotrophs via the secretion of alkaline phosphatases (APase) [16]. A recent study has suggested that the presence of CYN causes the secretion of alkaline phosphatase (APase) by other phytoplankton, thus increasing the amount of inorganic phosphate available to the CYN producer, and allowing it to out-compete other species in environments with limited inorganic phosphate [23]. The study showed that the addition of *Aphanizomenon ovalisporum* spent media containing high levels of CYN or purified CYN to various cultures of phytoplanktons, including *Chlamydomonas reinhardtii*, induced the upregulation of genes typically associated with limited inorganic phosphate and a rise in extracellular APase activity, despite high concentrations of inorganic phosphates present in the media [23]. The results were also supported by the field results showing a strong correlation between the abundance of CYN-producing *A. ovalisporum* and high APase activity in Lake Kinneret, Israel with enzyme-labeled fluorescence (ELF-APase) showing APase in various phytoplankton species, but not in *Aphanizomenon* [23]. Another study has also reported the increase in APase during the presence of *Aphanizomen ovalisporum* [67]. 

This strategy of using CYN to cause other organisms to overproduce APase, instead of producing the enzyme itself may be advantageous (in the evolutionary sense) because the cost to the cyanobacterium, in units of nitrogen and energy, have been estimated to be half the nitrogen cost of making APase, and less than the energy needed for the ribosomal synthesis of acid phosphatase [16,23]. 

Other studies have shown that concentrations of cyanotoxins increase when exposed to limited nutrients. Kurmayer [68] found that a *Nostoc* sp. also produced the highest concentrations of the toxin MCunder severe growth limiting conditions, due to phosphate (P-PO_4_) limitation. Oh *et al.* [69] showed that *Microcystis aeruginosa* increased MC when P was limited. The dinoflagellate *Alexandrium tamarense* increased STX production three- to four-fold under a P-limited environment [70]. Toxin production in the dinoflagellates *Gymnodinium catenatum* and *Alexandrium excavatum*, as well as the diatom *Pseudonitzschia multiseries*, have also been shown to increase under P stress [70,71]. Wang *et al.* [72] has postulated that APase might have some relationship with MC production in algal cells, as toxic *M. aeruginosa* enhanced APase activity when exposed to P-limiting conditions, whereas non-toxic forms did not. There is thus a possibility that these toxins may serve a role similar to that proposed for CYN, in terms of assisting with favourable nutrient dynamics, yet no studies have explored this to date: Raven [16] has suggested further topics for study in this field. 

Where cyanotoxins can be shown to mediate the nutrient dynamics for other organisms, this can be considered a form of indirect allelopathy [60]. However, in examples where cyanotoxins are able to influence nutrient availability (accessibility) to their producer species, the label of “physiological aide” may be more appropriate. Hence, this is a good example of the blurred line where one particular strategy (e.g., producing toxin that effectively delegates the metabolic cost of APase production to another competitor) could be considered both a form of allelopathy as well as a physiological aide. 

#### 3.3.2. Iron Scavenging

A number of studies have suggested the role of MC as an iron-scavenging molecule [17,73,74]. Iron is an essential nutrient for chlorophyll-a synthesis, respiration, photosynthesis, and nitrogen fixation within cyanobacteria. However at circumneutral pH, iron is generally limited. This has implications for water managers as most freshwater storages, which are at risk of toxic blooms, have a pH that is close to circumneutral or slightly alkaline. It has been shown that MC-producing strains of cyanobacteria possess more efficient Fe uptake systems compared with strains that do not produce MC [17,73]. It has been suggested that MC acts as an iron chelator inside the cell and is responsible for inactivating free cellular iron. It has also been suggested that MC production is regulated by the amount of free Fe in the cell [17]. This has recently been shown with iron-deficient media causing an increase in transcription of the *mcyD* gene responsible for the production of MC and an increase in MC levels [75]. However, Alexova *et al.* [74] found that transcription of some MC producing genes such as mcyA and mcyH did not increase with a decrease in iron, whereas MC concentrations did. Alexova and colleagues proposed that toxin production appears to give an advantage to MC-producing cyanobacteria in the early stages of exposure to severe iron stress and may protect the cell from reactive oxygen species-induced damage [74].

#### 3.3.3. Oxidative Stress and/or Carbon-Nitrogen Metabolism

A role for MC in relation to increasing survivorship to oxidative stress has been proposed [76]. A number of studies have shown an increase in transcription of *mcy* genes under conditions that promote oxidative stress, such as high light and iron deficient conditions [26,75,77]. However, rises in transcription of the *mcy* genes have not been linked with increase in toxin production: Rather, declining concentrations of MC are often noted [26,75,76,77]. The loss of MC from solution has been ascribed to binding of the toxin to various proteins [76]. Recently, Zilliges *et al.* [76] have shown that MC actively binds to proteins that are sensitive to redox changes, and that this binding is strongly increased under high light and oxidative stress conditions. Zilliges *et al.* [76] showed that MC-deficient mutants were more susceptible to high light and oxidative stress conditions. This suggests that MC may play an important role in the acclimation of toxin-producers to conditions involving oxidative stress. Zilliges *et al.* [76] also showed a link between MC and proteins involved in carbon metabolism, in particular the Calvin-Benson cycle. Alexova *et al.* [78] reported differences in the proteomes of six toxic and non-toxic strains of *Microcystis aeruginosa* and found that nine proteins were differentially expressed amongst the toxic and non toxin strains, with these proteins linked to carbon-nitrogen metabolism and redox maintenance. Neilan *et al.* [7] proposes that the studies by Zilliges *et al.* [76] and Alexova *et al.* [78] give support to a global role for MC in carbon-nitrogen metabolism and in redox control and/or perception of redox changes.

MC was also shown to accumulate when *Microcystis aeruginosa* PCC 7806 was exposed to low inorganic carbon conditions, and that the MC-deficient mutant was shown also to have a decreased ability to adapt to limited inorganic carbon compared to the MC-producer [79]. This result of dominance of the MC-producer over the MC-deficient mutant was also shown recently by Van de Waal *et al.* [80]. Jahnichen *et al.* [79] suggested a possible role of MC in enhancing the efficiency of the photosynthetic apparatus to adapt to changes in inorganic carbon [79]. Neilan *et al.* [7] also suggested a link between toxin production and photosynthesis as it appears that the regulation of toxin genes and toxin production by light appears to be universal among cyanobacteria.

El-Shehawy *et al.* [81] suggested that Nodularin (NOD), like MC, may have the ability to bind to proteins under stress conditions and may have a role in protein protection; given that, Jonasson *et al.* [82] showed that increased expression of the gene cluster for NOD production did not lead to increased levels of NOD produced. Neilan *et al.* [7] also suggest that the regulation of intracellular CYN may also occur at the protein level due to lack of correlation between transcript abundance of the cyr cluster and toxin concentration. In summary, the published information to date shows that toxins may have a role in improving the survivorship of toxin-producing algae through changes to oxidative stress responses and/or carbon-nitrogen metabolism, but the specific nature of this is not known. 

#### 3.3.4. Maintenance of Homeostasis

STX has been linked to the maintenance of Na^+^ homeostasis in cyanobacteria. Pomati *et al.* [83] showed that cyanobacteria (*C. raciborskii* and *A. circinalis*) capable of producing STX were able to prevent cell lysis caused by the sodium pump inhibitors veratridine (VTD) and vanadates (VAN), whereas complete cell lysis was shown for non-toxin producing strains. The addition of STX to the culture before treatment with VTD plus VAN prevented cell lysis of the same non-toxin producing strains. STX was also shown to ameliorate the adverse effect of VTD on metabolism. In another study, increases in Na^+^ stress have been shown to cause changes in STX production, with STX accumulation occurring at high intracellular Na^+^ levels [19]. The addition of lidocaine (a Na^+^ pump inhibitor) was also shown to lead to an accumulation of STX, whereas in the presence of amiloride, a Na^+^ blocker, STX accumulation did not occur [19,83,84]. This also provides support that STX may play a functional role in blocking Na^+^ channels in cyanobacteria to decrease sodium stress and maintain Na^+^ homeostasis. Recently Soto-Liebe *et al.* [85] have shown that STX is exported out of the cyanobacteria (*Raphidiopsis brookii*) in response to increases in the cations Na^+^ and K^+^. This finding also provides support for the possible role of STX in ensuring homeostasis against variations in salinity. When examining the mechanism by which STX toxin provides toxicity to eukaryotes by blocking Na^+^ channels, leading to decrease in flow of sodium ions which causes an inhibition of neuronal transmission [86], it is not unreasonable to propose that the ecological role for STX may be similar. If the role of STX is to limit Na^+^ uptake under conditions of high pH or salt stress, then this may also provide the STX producers with a competitive advantage over non-STX producing species, thus helping to explain the presence of STX in a range of aquatic environments from marine to freshwater. 

Although there is currently no evidence for the role of anatoxin-a in Na^+^ homeostasis, this remains a possibility. Anatoxin-a causes toxicity in vertebrates by binding to the nicotinic receptor on sodium channels, causing the channels to open allowing sodium ions to flow in, inducing over stimulation of the nerve and muscle cells leading to paralysis [86]. Anatoxin-a may therefore play an opposite role to STX in maintaining Na^+^ homeostasis. At low Na^+^ levels, anatoxin-a may play a role in sodium uptake by opening up the cell’s sodium channels. The ability to produce both STX and anatoxin-a has been recorded for a number of genera of cyanobacteria (*Anabaena*, *Aphanizomenon*, *Cylindrospermopsis*, *Planktothrix*) and it is possible that both these cyanotoxins are produced to help maintain sodium homeostasis at different environmental scales (high Na^+^ (STX), low Na^+^ (anatoxin-a)). Another possible role is that anatoxin-a and/or STX may be used to disrupt the Na^+^ homeostasis of other organisms as a type of defensive or allelopathic strategy.

#### 3.3.5. Roles as Infochemicals

Toxins produced by cyanobacteria may act as info-chemicals or signalling molecules. A study by Schatz *et al.* [87] showed that when exposed to MC, *Microcystis* cells increased the accumulation of McyB and enhanced the production of microcystins within themselves. The author proposes that the lysis of a fraction of the *Microcystis* population is sensed by the rest of the cells via the release of non-ribosomal peptides (e.g., microcystin), allowing the remaining cells to respond by increasing their ecological fitness by raising their ability to produce toxic compounds [87]. 

MC has also been linked with a role in signalling colony formation and aiding in its production [18,88,89]. Gan *et al.* [18] showed that MC’s significantly increased colony sizes of a number of *Microcystis* sp. Decreases in extracellular MC through microbially-driven degradation also caused the colonies to decrease in size: This suggests that MC is needed to maintain colony size. Kurmayer *et al.* [90] also found that larger colonies of *Microcystis* in a lake in Berlin contained higher proportions of MC-producers than did small colonies. MC also causes increased production of extracellular polysaccharides (EPS) and the up-regulation of four polysaccharide biosynthesis-related genes: *capD*, *csaB*, *tagH*, and *epsL*. EPS are associated with cell aggregates; they are found mainly in the mucilage or the sheath of the microorganism and affect the stickiness of the cell surface, thus allowing colony formation in some algal species. The polysaccharide layer of the cell was also shown to become thicker in response to MC [18]. EPS production has been linked with stressful conditions, such as predation and adverse environmental conditions. The ability of MC to trigger EPS and increase the formation of colonies may provide MC-producers with an advantage against grazing and stressful conditions [18]. 

## 4. Understanding the Role of Toxins: A Whole-of-System Approach

Most research effort to date has focused on understanding the physico-chemical conditions under which toxin production is triggered. However, it appears that a much more comprehensive and holistic “systems approach” is needed to begin understanding how the complex mix of environmental conditions, resource competition, and cell physiology interact with each other to encourage toxigenicity and stimulate toxin production. For example, the overall message from the analysis of abiotic components is that whilst toxin production often appears to be decoupled from cell growth rates in the purely quantitative sense, there is a *de facto* relationship between those conditions that regulate cellular growth rates, and those that encourage maximal toxin production. Here, one or more environmental conditions either in the sub-optimal or supra-optimal range for cell reproduction. The impacts of these conditions on the growth and/or survivorship of the species are retarded population growth and/or death (in the extreme cases). At the cellular level, the potential response to this is twofold: To increase toxin production with the objective of influencing the external environment; or to reduce toxin production, with the benefit being reduced metabolic demand and the potential to re-route that investment internally, in order to maintain or accelerate cell growth. 

For the biotic components, the two main schools of thought appear to be the possible role of toxins in conferring direct competitive advantage, through either grazing defence or allelopathy; or, an internal role for toxins in terms of cellular physiology, and the ability of cells to access and utilize the resources for growth. The latter “physiological aide” argument also provides benefit, but does so indirectly by mediating other cell processes. 

With respect to a role for toxins in grazing defence, the pressures on producer species could occur from protozoans, zooplankton, or higher-level organisms (invertebrates, vertebrates). This produces a threat of reduced cell number or—in extreme cases—localized extinction of the population. A suite of possible responses is known from cyanobacteria, of which toxin production is only one. Similarly, the pressure of resource competition from other primary producers, principally for light and nutrients, also leads to a state of population threat for cyanobacteria: Here, toxins may be produced as allelopathic compounds. As described above, cyanotoxins have also been shown to help mediate nutrient uptake, assist in photosynthetic efficiency (through iron scavenging), protect against oxidative stress, maintain homeostasis, and participate in cell-to-cell signalling. 

In the community context, there are essentially two schools of thoughts for cyanotoxins: Firstly, toxins can be regarded as environmental tolerance traits that allow the producer organisms to cope with abiotic environmental changes. In some cases, this may involve toxin production with the apparent self-limitation of cell growth, as discussed above: This would avoid a population explosion and subsequent bust. Secondly, cyanotoxins can be linked with resource acquisition/enemy resistance traits: This provides an advantage over competitors and/or predators by exploiting common limiting resources or conveying resistance. Regardless, both these approaches are likely to lead to a situation of increased dominance of toxin-producing blue-green algae, in both niche and non-niche environments.

In considering the possible ecological roles for cyanotoxins, it is important to acknowledge that blue-greens (and their toxins) have persisted across considerable timescales, and the changing environments that have accompanied these. The earth’s environment at the time when cyanotoxins originally evolved was very different to the current environment, and evidence for this is reflected in contemporary studies: Consider, for example, the stability of CYN in boiling waters and extreme pH values [91]. The fact that toxin production has been retained over long periods—despite the metabolic cost—seems to suggest longevity in any competitive advantage conferred by the toxin. Were this not the case, then genes involved in toxin production would likely have been lost over time, and, in the absence of new environmental pressures, there would be a decrease in the abundance of toxigenic compared with non-toxigenic cyanobacteria. Rather, the reverse seems to be true, with an increased abundance of toxigenic blooms being reported in the literature; however, this may result from increased scientific observations and reporting, rather than being a specific rise. It is interesting to note that cyanotoxins have also been shown to affect mammals with no direct competitive or predatory link to algae (for example, several studies with mice and cattle). Again, this suggests that the evolutionary drivers for toxin production may have changed significantly since their emergence: Whether or not this “non-target” toxicity will eventually fall away is not known. It is also useful to note that almost all cyanobacterial toxins are produced through biosynthetic pathways, which are highly variable, because they are controlled by genetic and/or enzymatic factors. This provides a situation whereby the nature and composition of toxins produced by blue-greens is able to change rapidly in response to abiotic and biotic factors: This allows for the possibility of divergent (and more advantageous) ecological roles of toxins to be developed over time. 

## 5. Implications for Future Management

As global climate change progresses, a greater range of cyanotoxin producers are likely to expand into subtropical and temperate climes, and a greater breadth of aquatic species will become vulnerable to toxicity and toxin accumulation. Increasing eutrophication and creation of more storage impoundments are also factors that are likely to create an increased prevalence of toxin producers. Further conceptual and empirical research into the ecological role and environmental triggers of toxin production is needed to better understand the complex suite of conditions that influence the toxigenic capacity, as well as the level of toxin production, in blue-green algae. Necessarily, this needs to include not only the triggers for toxin production, but also for cessation of toxin production. Conceivably, information may come to light that would allow for new interventions that discourage toxigenic strains of algae: This would be particularly useful for drinking water storage impoundments. 

## 6. Concluding Thoughts

In summary, cyanotoxins appear to have evolved due to a combination of pressures from abiotic or biotic factors. There are two main themes underpinning their ecological roles: First, they evolved for the purposes of direct competitive advantage (e.g., through grazing defence mechanisms or to act as an allopathic compound against competitors); and/or second, they evolved to aide general physiological function. Notwithstanding either (or both) of these, it must also be recognised that the original ecological roles of cyanotoxins are also likely to have mutated or been lost over evolutionary time, possibly to be replaced with new functions. A number of research questions remain outstanding with respect to understanding the “why” of toxin production by the blue-green algae. Firstly, there are no published articles that examine why many blue-greens produce such a wide variety of toxins. For example, *Cylindrospermopsis* can produce multiple analogs of CYN, in addition to STX and anatoxins; and there are some 80+ variants known for MC. Simplistically, this could be viewed as a result of multiple pressures, but not enough is known to understand the specifics of this, including whether new forms of toxins may be expected in the coming years. Secondly, why do both toxigenic and non-toxigenic species of cyanotoxin producers exist, and seemingly with comparable success? What, if any, potential is there for non-toxic strains to take advantage of the production of toxins by co-occurring strains, without supporting the costs of synthesis? Are there indeed very few non-toxic strains, but the current state of research and analytical tools means that tests for many types of compounds do not yet exist? 

To obtain a clearer picture of whether or not toxicogenicity is likely to be retained as a feature of cyanobacteria into the future, work is needed on determining the synergistic effects of different abiotic and biotic factors on toxin production. Ideally, this would involve laboratory studies that are validated by field findings, and the use of molecular data such as the transcription of toxin gene clusters under the same conditions. 

In conclusion, the above review has suggested that there are likely to be a multiplicity of roles for cyanobacterial toxins: These vary with particular toxin, and with their producer species. Any given toxin may have one or more ecological functions, but assigning these with confidence is challenged by the existing state of the literature, which reflects the lack of systematic hypothesis testing for each compound and producer-species combination. 
